# Associations between maternal exposure to per- and polyfluoroalkyl substances and functional constipation in children at 3 and 4 years: the Japan Environment and Children’s Study

**DOI:** 10.1265/ehpm.25-00399

**Published:** 2026-05-21

**Authors:** Kohei Hasegawa, Yuji Inaba, Shoji Saito, Takumi Shibazaki, Margareta Laurence Pisonningtyas, Shoji F. Nakayama, Michihiro Kamijima, Teruomi Tsukahara, Tetsuo Nomiyama, the Japan Environment and Children’s Study (JECS) Group

**Affiliations:** 1 Department of Preventive Medicine and Public Health, Shinshu University School of Medicine, 3-1-1 Asahi, Matsumoto, Nagano 390-8621, Japan; 2 Center for Perinatal, Pediatric, and Environmental Epidemiology, Shinshu University School of Medicine, 3-1-1 Asahi, Matsumoto, Nagano 390-8621, Japan; 3 Department of Neurology, Nagano Children’s Hospital, 3100 Toyoshina, Azumino, Nagano 399-8288, Japan; 4 Life Science Research Center, Nagano Children’s Hospital, 3100 Toyoshina, Azumino, Nagano 399-8288, Japan; 5 Department of Pediatrics, Shinshu University School of Medicine, 3-1-1 Asahi, Matsumoto, Nagano 390-8621, Japan; 6 Faculty of Medicine, Diponegoro University, Jalan Prof. Soedarto, Tembalang, Kec. Tembalang, Kota Semarang, Jawa Tengah 50275, Indonesia; 7 Japan Environment and Children’s Study Programme Office, National Institute for Environmental Studies, 16-2 Onogawa, Tsukuba, Ibaraki 305-8506, Japan; 8 Department of Occupational and Environmental Health, Nagoya City University Graduate School of Medical Sciences, 1 Kawasumi, Mizuho-cho, Mizuho-ku, Nagoya, Aichi 467-8601, Japan; 9 Department of Occupational Medicine, Shinshu University School of Medicine, 3-1-1 Asahi, Matsumoto, Nagano 390-8621, Japan

**Keywords:** Maternal exposure, Per- and polyfluoroalkyl substances, Functional constipation, Birth cohort

## Abstract

**Background:**

Research over the past decade has indicated that exposure to per- and polyfluoroalkyl substances (PFAS) disrupts the intestinal barrier, potentially contributing to the development of gastrointestinal disorders. More recently, constipation has been hypothesized as one such outcome. However, epidemiological studies examining this association remain limited.

**Methods:**

We analyzed data from 17,686 mothers and their children from the Japan Environment and Children’s Study, a Japanese birth cohort study. We used maternal plasma samples collected during pregnancy to determine PFAS concentrations. Of the 28 PFAS compounds analyzed, we included seven compounds that were quantified in more than 80% of the samples. We assessed functional constipation in children at ages 3 and 4 using caregiver-completed questionnaires based on the Rome III criteria. Logistic regression models were used to examine associations between maternal PFAS concentrations and childhood functional constipation, applying a Bonferroni-corrected significance threshold of P < 0.0035 to account for multiple comparisons.

**Results:**

Functional constipation was observed in 11.3% of children at age 3 and 9.8% at age 4. After adjusting for potential confounders, we observed no significant associations between any of the seven maternal PFAS concentrations and functional constipation in children at 3 years of age. However, at 4 years, perfluorooctanoic acid (PFOA) concentrations were significantly associated with functional constipation, with an adjusted odds ratio of 1.13 (95% confidence interval: 1.05–1.21, P = 0.00057) per doubling of concentration. The remaining six PFAS compounds showed no significant associations at 4 years.

**Conclusions:**

The single positive association between maternal PFOA concentration and functional constipation at 4 years of age is unlikely to reflect a true causal relationship, given limited biological plausibility. Our results do not provide evidence of an association between prenatal PFAS exposure and functional constipation in children, although further investigation is warranted.

**Supplementary information:**

The online version contains supplementary material available at https://doi.org/10.1265/ehpm.25-00399.

## 1. Background

Functional constipation is defined as constipation without an identifiable organic etiology and is characterized by symptoms including reduced defecation frequency, hard stools, and abdominal pain [1]. This condition accounts for more than 95% of total constipation cases [2]. A meta-analysis reported a pooled prevalence of 9.5% among children worldwide [3], making it a public health concern. While several associated factors, such as dietary habits, psychological distress, and socioeconomic status, have been identified [3, 4], limited research has examined the role of environmental exposure in this condition [5–7].

Per- and polyfluoroalkyl substances (PFAS) are synthetic chemicals widely used in industrial and consumer applications [8]. The persistence of PFAS in the environment and the human body can lead to bioaccumulation, accounting for the ubiquitous detection of these substances in human populations [9]. PFAS readily cross the placental barrier, resulting in direct fetal exposure [10]. Epidemiological evidence suggests that prenatal PFAS exposure may be associated with health outcomes in children [11, 12].

Research over the past decade indicates that PFAS may alter gut microbiota and impair intestinal barrier function, potentially contributing to the development of gastrointestinal disorders [13]. Recently, PFAS exposure has been hypothesized to contribute to constipation [7]. To date, only one cross-sectional study—a population-based analysis using National Health and Nutrition Examination Survey (NHANES) data from U.S. adults—has examined potential associations between PFAS exposure and constipation [7]. That study reported an inverse association, suggesting a protective role for PFAS. To the best of our knowledge, no previous studies have examined whether prenatal PFAS exposure is associated with constipation in children.

To address this knowledge gap, this study examined whether maternal PFAS exposure during pregnancy is associated with functional constipation in children by analyzing data from a birth cohort study in Japan.

## 2. Methods

### 2.1. Study population

The Japan Environment and Children’s Study (JECS) is an ongoing prospective birth cohort study in Japan. Recruitment took place from January 2011 to March 2014 across selected research regions in Japan, officially termed “Study Areas.” Eligible participants were pregnant women who (a) resided within a Study Area at enrollment, (b) had expected delivery dates after 1 August 2011, and (c) had sufficient Japanese language proficiency to complete the study’s self-reported questionnaires. Details on the cohort recruitment and baseline profiles have been reported previously [14, 15]. For the present analysis, datasets containing PFAS measurements were downloaded on 1 June 2025 from the JECS website for affiliated researchers.

In JECS, PFAS measurements of maternal blood samples were conducted on a subset of approximately 25,000 participants from the total cohort due to budgetary constraints [16, 17]. Participants for PFAS analysis were selected through a two-step process. First, all participants enrolled in the Sub-Cohort Study of JECS (approximately 5,000 participants) were selected. The Sub-Cohort Study is a detailed investigation conducted on a subset of JECS participants [18]. Second, approximately 20,000 additional participants were randomly selected from those not enrolled in the Sub-Cohort Study.

**Fig. 1:**
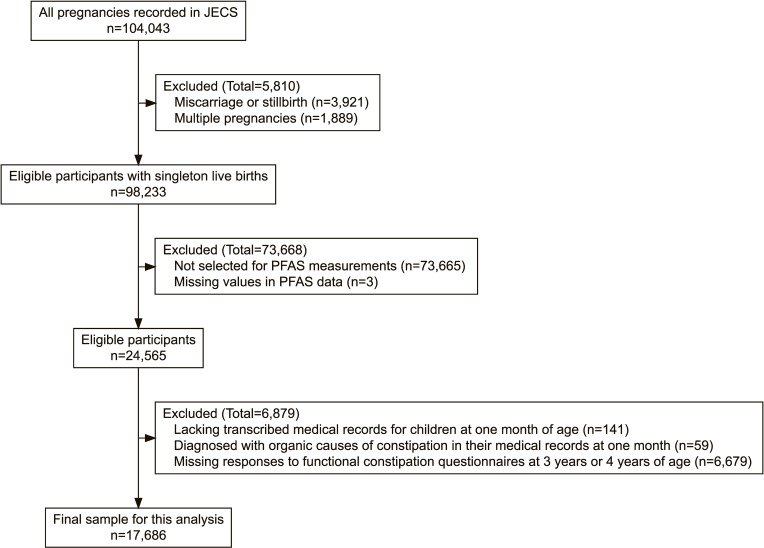
Participant selection flowchart for the present analysis. JECS, Japan Environment and Children’s Study; PFAS, per- and polyfluoroalkyl substances.

The final sample included 17,686 participating mothers and their children from JECS (Fig. 1). From all recorded pregnancies, we initially excluded miscarriages, stillbirths, and multiple pregnancies, leaving only singleton live births. We then excluded participants not selected for PFAS measurement and those with missing values in PFAS data. We further excluded participants lacking transcribed medical records for their children at one month of age, as well as those whose children had documented diagnoses of organic causes of constipation in their medical records at one month—specifically, congenital hypothyroidism, anorectal anomaly, Hirschsprung’s disease, spina bifida, or trisomy 21. Subsequently, we excluded participants who lacked responses to the functional constipation questionnaires at 3 years or 4 years of age.

### 2.2. Maternal blood PFAS concentrations

Medical staff collected plasma samples for PFAS determination. Sample collection times (gestational weeks) were distributed as follows: minimum, 7.1 weeks; 2.5th percentile, 10.7; 5th percentile, 11.4; 10th percentile, 12.1; median (50th percentile), 15.7; 90th percentile, 20.3; 95th percentile, 21.0; 97.5th percentile, 21.7; and maximum, 35.9. Analytical methods for determining PFAS concentrations in plasma samples, including quality assurance and quality control procedures, have been described previously [16, 17, 19]. In brief, the process involved protein precipitation, automated solid-phase extraction pretreatment, and column-switching liquid chromatography-tandem mass spectrometry. The lowest concentration minimum reporting level (LCMRL) and method detection limit were established following U.S. Environmental Protection Agency procedures [20].

We measured 28 PFAS compounds (Supplementary Table S1), but focused on seven compounds that exceeded the LCMRLs in more than 80% of samples: perfluorooctanoic acid (PFOA), perfluorononanoic acid (PFNA), perfluorodecanoic acid (PFDA), perfluoroundecanoic acid (PFUnA), perfluorotridecanoic acid (PFTrDA), perfluorohexane sulphonic acid (PFHxS), and perfluorooctane sulphonic acid (PFOS). We determined both total and linear isomer concentrations for compounds with branched isomers. Due to the strong correlation between the two concentrations [16], we focused our analysis on linear isomer concentrations. We used a Gibbs sampler-based imputation method for left-censored missing values in regression analyses to handle PFAS concentrations below the LCMRLs [21].

### 2.3. Functional constipation in children

Functional constipation was assessed using caregiver-completed questionnaires based on the Japanese translation of the Rome III criteria [22, 23], which were administered when children were approximately 3 and 4 years old. Caregivers indicated “yes” or “no” to whether the following six symptoms had persisted for more than one month: (a) two or fewer defecations per week; (b) at least one episode per week of incontinence after the acquisition of toileting skills; (c) history of excessive stool retention; (d) history of painful or hard bowel movements; (e) presence of a large fecal mass in the rectum; and (f) history of large-diameter stools that may obstruct the toilet. Functional constipation was defined as a “yes” response to two or more items.

### 2.4. Confounders and other variables

We initially constructed a directed acyclic graph based on a review of the relevant literature (Supplementary Fig. S1). Using the DAGitty web application (https://dagitty.net/), we identified the following variables as the minimal sufficient set of confounders: maternal age at childbirth, maternal body mass index before pregnancy, parity, maternal smoking, maternal education, household income, maternal dietary habits, and Study Area at recruitment. Models were adjusted for this minimal sufficient set throughout the analyses unless otherwise specified. For sensitivity analyses, we also examined models that were additionally adjusted for cesarean birth, breastfeeding duration, or sex of the child. In further sensitivity analyses, we examined models with additional adjustment for daycare attendance and diaper use during sleep at the time of outcome assessment.

Data collection and variable definitions were as follows. Maternal body mass index before pregnancy was transcribed from medical records but supplemented by self-reports. We further categorized body mass index using the 18.5 and 25.0 kg/m^2^ cutoffs. Parity was transcribed from medical records before pregnancy and categorized as primiparous or multiparous. Cesarean birth status was also transcribed from medical records.

Maternal smoking, maternal education, household income, and maternal dietary habits were determined by the responses to the self-reported questionnaires. JECS administered these questionnaires at two time points during pregnancy: the first at a median gestational age of 14.4 weeks and the second at 27.0 weeks. Maternal smoking was assessed at the first time point with response options including “never,” “previously did, but quit before realizing current pregnancy,” “previously did, but quit after realizing current pregnancy,” and “currently smoking.” We regrouped these responses into smoking or not smoking during pregnancy. Maternal education was included in the self-reported questionnaire at the second time point with possible responses of “junior high school,” “high school,” “technical junior college,” “technical/vocational college,” “associate degree,” “bachelor’s degree,” and “graduate degree (master’s/doctor’s).” We regrouped them based on the duration of education [24]: ≤12 years (encompassing the first two categories), 13–15 years (third through fifth categories), and ≥16 years (final two categories). Household income was also assessed in the questionnaire at the second time point, with nine possible response categories: <2, 2 to <4, 4 to <6, 6 to <8, 8 to <10, 10 to <12, 12 to <15, 15 to <20, and ≥20 million yen per year. These were regrouped into five categories: <2, 2 to <4, 4 to <6, 6 to <8, and ≥8 million yen per year.

Maternal dietary habits were evaluated using a food frequency questionnaire [25], administered at the first of the two pregnancy time points described above. The questionnaire evaluated dietary patterns, including the frequency and portion sizes of selected foods, over the preceding year. Daily intake of food groups was calculated from questionnaire responses using the Standard Tables of Food Composition in Japan 2010 [26]. Among the food groups, we estimated daily intake of seafood as a known source of PFAS exposure [27–29], along with other potential dietary sources, including eggs, meat, fruit, and vegetables [30]. Daily intake estimates for each of the five food groups were categorized into quartiles separately, without summing or other computations, and they were simultaneously included as five covariates in the regression models, to serve as proxies for maternal dietary habits.

Breastfeeding duration was assessed through a caregiver-reported questionnaire when the child was one year of age. The questionnaire required caregivers (mostly mothers) to indicate whether the child was breastfed during each month from the first month to 12 months after birth. Breastfeeding duration was defined as the final month of breastfeeding within the 12 months and subsequently categorized as <7 or ≥7 months [31].

Daycare attendance and diaper use during sleep at ages 3 and 4 years were assessed using caregiver-reported questionnaires administered at these ages. These were the same questionnaires used to define functional constipation at ages 3 and 4 years. Caregivers indicated whether their child attended any form of childcare (preschool, daycare center, or nursery school) by selecting “yes” or “no,” with affirmative responses classified as daycare attendance. Diaper use during sleep was assessed in the same manner, with responses of “yes” or “no.”

### 2.5. Statistical analyses

Statistical analyses were performed using Python 3 (version 3.8.10) and R (versions 3.6.3 and 4.1.1). Statistical significance was defined as P < 0.05 (two-sided). To account for multiple comparisons, Bonferroni correction was applied with a significance threshold of P < 0.05/(7 PFAS × 2 outcomes) = 0.05/14 = 0.0035 for the primary analyses and corresponding sensitivity analyses. Bonferroni correction was not applied to subsequent analyses.

Logistic regression models were used to examine the association between maternal blood PFAS concentrations and functional constipation at each time point. Unless otherwise noted, we fit single-pollutant logistic regression models, each including one PFAS concentration. PFAS concentrations were logarithmically transformed using base 2 [17]. We presented associations as odds ratios per doubling in each PFAS concentration with their 95% confidence intervals (CIs). We included the aforementioned minimal sufficient set for confounder adjustment. We handled missing values by multiple imputation with chained equations [32]. We generated a total of 10 imputed datasets and combined the results using Rubin’s rule.

We also conducted several supplementary analyses. To examine the exposure–response relationships, PFAS concentrations were categorized into quartiles, and thin-plate smoothing splines were applied using the mgcv package in R [33]. To account for potential confounding by co-exposure to other PFAS, we developed multi-pollutant models including all seven PFAS as separate predictors. Using these same multi-pollutant models, we estimated the joint effect of all seven compounds using an additive main-effects approach, which sums the individual estimates for each PFAS [34]. Stratified analyses were conducted by child sex, a recognized effect modifier of environmental exposures [35]. Additional stratified analyses were performed by mode of delivery and breastfeeding status, as prior research suggests these factors may modify associations between PFAS exposure and infant microbiome diversity [36]. We formally assessed differences between strata by calculating P values using a previously described method [37], with statistical significance defined as P < 0.05.

In addition to the supplementary analyses described above, we performed several sensitivity analyses to assess the robustness of our findings. First, we used functional constipation at both 3 and 4 years as a combined outcome, instead of treating each time point as a separate outcome [38]. Second, we examined models additionally adjusted for child sex, cesarean birth, or breastfeeding duration. Third, we additionally adjusted for daycare attendance and diaper use during sleep at the time of outcome assessment as proxies for social environments relevant to constipation development [39]. Fourth, we tested models without adjustment for dietary habits. Fifth, to address potential variation in sampling time, we excluded participants whose blood samples for PFAS measurement were collected in the earliest and latest 2.5% of gestational weeks. We also tested an alternative approach that excluded the earliest and latest 5% or 10%. Finally, given the relatively common occurrence of outcomes (∼10%), we applied modified Poisson regression models in place of logistic regression [40].

## 3. Results

### 3.1. Descriptive analysis

Most mothers were under 35 years of age, had a body mass index ranging from 18.5 kg/m^2^ to 24.9 kg/m^2^, were multiparous, and reported no smoking during pregnancy (Table 1). The prevalence of functional constipation was 11.3% at 3 years of age and 9.8% at 4 years, with 4.1% of children experiencing functional constipation at both time points. Baseline characteristics showed minor differences between the included and excluded groups, although the included group had slightly more years of education. Among the seven PFAS compounds included in this analysis, the highest median concentration was observed for PFOS, at 3.0 ng/mL (Table 2). Spearman’s correlation coefficients for the seven PFAS compounds ranged from 0.10 (PFTrDA and PFHxS) to 0.85 (PFUnA and PFTrDA) (Supplementary Table S2).

**Table 1:** Characteristics of participants, comparing included and excluded groups

**Characteristics**	**Variable**	**Included** **(n = 17,686)**	**Excluded** **(n = 80,547)**
Maternal	Age at childbirth		
	<35 years	12,613 (71.3)	58,843 (73.1)
	≥35 years	5,073 (28.7)	21,695 (26.9)
	Missing	0	9
	Body mass index before pregnancy		
	<18.5 kg/m^2^	2,798 (15.8)	13,069 (16.2)
	18.5–24.9 kg/m^2^	13,135 (74.3)	58,650 (72.9)
	≥25.0 kg/m^2^	1,745 (9.9)	8,710 (10.8)
	Missing	8	118
	Parity		
	0	5,447 (31.0)	23,944 (30.0)
	≥1	12,099 (69.0)	55,930 (70.0)
	Missing	140	673
	Smoking during pregnancy		
	No	16,972 (96.7)	74,569 (94.8)
	Yes	588 (3.3)	4,065 (5.2)
	Missing	126	1,913
	Education in years		
	≤12 years	5,671 (32.3)	29,196 (37.3)
	13–15 years	7,604 (43.3)	32,709 (41.7)
	≥16 years	4,273 (24.4)	16,457 (21.0)
	Missing	138	2,185
	Household income		
	<2,000,000 yen/year	736 (4.4)	4,351 (6.0)
	2,000,000–3,999,999 yen/year	5,544 (33.3)	25,424 (34.9)
	4,000,000–5,999,999 yen/year	5,704 (34.3)	23,903 (32.8)
	6,000,000–7,999,999 yen/year	2,794 (16.8)	11,454 (15.7)
	≥8,000,000 yen/year	1,858 (11.2)	7,814 (10.7)
	Missing	1,050	7,601
	Seafood intake in early pregnancy		
	Q1: 0.0–16.9 g/day	4,197 (23.8)	19,903 (25.1)
	Q2: 17.0–31.3 g/day	4,453 (25.2)	19,891 (25.1)
	Q3: 31.4–50.5 g/day	4,576 (25.9)	19,599 (24.7)
	Q4: 50.6–3,500.5 g/day	4,422 (25.1)	19,861 (25.1)
	Missing	38	1,293
	Egg intake in early pregnancy		
	Q1: 0.0–10.6 g/day	1,596 (9.0)	7,865 (9.9)
	Q2: 10.7–24.9 g/day	4,499 (25.5)	20,317 (25.6)
	Q3: 25.0–39.2 g/day	5,967 (33.8)	26,102 (32.9)
	Q4: 39.3–600.0 g/day	5,586 (31.7)	24,970 (31.5)
	Missing	38	1,293
	Meat intake in early pregnancy		
	Q1: 0.0–39.0 g/day	4,414 (25.0)	19,785 (25.0)
	Q2: 39.1–61.5 g/day	4,571 (25.9)	19,668 (24.8)
	Q3: 61.6–93.3 g/day	4,508 (25.5)	19,707 (24.9)
	Q4: 93.4–6,050.0 g/day	4,155 (23.5)	20,094 (25.4)
	Missing	38	1,293
	Fruit intake in early pregnancy		
	Q1: 0.0–49.4 g/day	4,164 (23.6)	20,046 (25.3)
	Q2: 49.5–116.2 g/day	4,609 (26.1)	19,610 (24.7)
	Q3: 116.3–209.6 g/day	4,591 (26.0)	19,653 (24.8)
	Q4: 209.7–7,939.1 g/day	4,284 (24.3)	19,945 (25.2)
	Missing	38	1,293
	Vegetable intake in early pregnancy		
	Q1: 0.0–98.9 g/day	4,297 (24.3)	19,922 (25.1)
	Q2: 99.0–156.8 g/day	4,384 (24.8)	19,846 (25.0)
	Q3: 156.9–238.3 g/day	4,435 (25.1)	19,788 (25.0)
	Q4: 238.4–5,740.8 g/day	4,532 (25.7)	19,698 (24.9)
	Missing	38	1,293
	Cesarean birth		
	No	14,498 (82.2)	65,003 (80.9)
	Yes	3,148 (17.8)	15,327 (19.1)
	Missing	40	217
	Breastfeeding duration		
	<7 months	3,798 (21.8)	17,569 (24.7)
	≥7 months	13,647 (78.2)	53,641 (75.3)
	Missing	241	9,337
	Study Area at recruitment		
	Hokkaido	1,414 (8.0)	6,339 (7.9)
	Miyagi	1,578 (8.9)	7,360 (9.1)
	Fukushima	2,124 (12.0)	10,429 (12.9)
	Chiba	952 (5.4)	4,853 (6.0)
	Kanagawa	1,040 (5.9)	5,267 (6.5)
	Koshin	1,247 (7.1)	5,787 (7.2)
	Toyama	1,118 (6.3)	4,200 (5.2)
	Aichi	1,053 (6.0)	4,366 (5.4)
	Kyoto	711 (4.0)	3,103 (3.9)
	Osaka	1,429 (8.1)	6,213 (7.7)
	Hyogo	875 (4.9)	4,089 (5.1)
	Tottori	529 (3.0)	2,429 (3.0)
	Kochi	1,130 (6.4)	5,657 (7.0)
	Fukuoka	1,411 (8.0)	5,921 (7.4)
	Minami Kyushu/Okinawa	1,075 (6.1)	4,534 (5.6)
Child	Sex		
	Female	9,098 (51.4)	41,243 (51.2)
	Male	8,588 (48.6)	39,293 (48.8)
	Missing	0	11
	Daycare attendance at 3 years		
	No	6,366 (37.1)	22,528 (36.8)
	Yes	10,789 (62.9)	38,680 (63.2)
	Missing	531	19,339
	Diaper use during sleep at 3 years		
	No	2,414 (13.7)	8,138 (12.9)
	Yes	15,249 (86.3)	54,927 (87.1)
	Missing	23	17,482
	Functional constipation at 3 years		
	No	15,683 (88.7)	50,866 (88.2)
	Yes	2,003 (11.3)	6,808 (11.8)
	Missing	0	22,873
	Daycare attendance at 4 years		
	No	1,061 (6.4)	3,952 (7.1)
	Yes	15,438 (93.6)	51,353 (92.9)
	Missing	1,187	25,242
	Diaper use during sleep at 4 years		
	No	9,601 (54.5)	31,060 (53.0)
	Yes	8,007 (45.5)	27,590 (47.0)
	Missing	78	21,897
	Functional constipation at 4 years		
	No	15,951 (90.2)	51,640 (89.5)
	Yes	1,735 (9.8)	6,058 (10.5)
	Missing	0	22,849
	Functional constipation at both 3 and 4 years		
	No	16,963 (95.9)	48,757 (95.9)
	Yes	723 (4.1)	2,059 (4.1)
	Missing	0	29,731

Note: Data are presented as n (%). All percentages are calculated by excluding missing values.

**Table 2:** Distribution of maternal blood PFAS concentrations (ng/mL) among participants included in this analysis

**PFAS**	**Minimum**	**25th** **percentile**	**Median**	**75th** **percentile**	**Maximum**
PFOA	<LCMRL	1.1	1.6	2.5	45
PFNA	<LCMRL	1.0	1.4	1.9	36
PFDA	<LCMRL	0.38	0.50	0.69	12
PFUnA	<LCMRL	0.85	1.1	1.5	11
PFTrDA	<LCMRL	0.18	0.26	0.36	2.0
PFHxS	<LCMRL	0.23	0.33	0.48	7.2
PFOS	<LCMRL	2.1	3.0	4.1	39

Note: LCMRL, lowest concentration minimum reporting level; PFAS, per- and polyfluoroalkyl substances; PFDA, perfluorodecanoic acid; PFHxS, perfluorohexane sulphonic acid; PFNA, perfluorononanoic acid; PFOA, perfluorooctanoic acid; PFOS, perfluorooctane sulphonic acid; PFTrDA, perfluorotridecanoic acid; PFUnA, perfluoroundecanoic acid.

### 3.2. Primary analyses

No significant associations were identified between any of the seven PFAS concentrations and functional constipation at 3 years of age. When functional constipation at 4 years of age was used as the outcome measure, PFOA concentrations demonstrated a significant positive association with an adjusted odds ratio of 1.13 (95% CI: 1.05–1.21, P = 0.00057) per doubling of concentration (Table 3). No associations were observed for the remaining six PFAS compounds.

**Table 3:** Associations between maternal PFAS concentrations and functional constipation in children

		**Crude**		**Adjusted**	

**Outcome** **assessment**	**PFAS**	**Odds ratio (95% CI)**	**p**	**Odds ratio (95% CI)**	**p**
At 3 years	PFOA	1.05 (1.00–1.10)	0.0541	1.06 (0.99–1.13)	0.0822
	PFNA	0.98 (0.92–1.05)	0.6165	1.00 (0.93–1.08)	0.9009
	PFDA	0.96 (0.90–1.02)	0.1664	0.99 (0.92–1.07)	0.8763
	PFUnA	0.92 (0.86–0.99)	0.0210	0.96 (0.89–1.04)	0.3299
	PFTrDA	0.98 (0.95–1.02)	0.3997	1.01 (0.97–1.05)	0.7515
	PFHxS	1.05 (1.00–1.10)	0.0317	1.05 (1.00–1.11)	0.0504
	PFOS	0.97 (0.91–1.04)	0.4430	1.01 (0.93–1.09)	0.8207
At 4 years	PFOA	1.11 (1.05–1.17)	0.00020	1.13 (1.05–1.21)	0.00057
	PFNA	1.03 (0.96–1.11)	0.3919	1.03 (0.95–1.12)	0.4195
	PFDA	0.94 (0.88–1.00)	0.0680	0.95 (0.88–1.03)	0.2013
	PFUnA	0.88 (0.81–0.95)	0.00070	0.88 (0.81–0.96)	0.0049
	PFTrDA	0.93 (0.89–0.97)	0.00023	0.94 (0.90–0.98)	0.0045
	PFHxS	1.06 (1.01–1.11)	0.0219	1.05 (0.99–1.11)	0.0794
	PFOS	0.94 (0.88–1.01)	0.0778	0.94 (0.86–1.02)	0.1394

Note: PFAS, per- and polyfluoroalkyl substances; PFDA, perfluorodecanoic acid; PFHxS, perfluorohexane sulphonic acid; PFNA, perfluorononanoic acid; PFOA, perfluorooctanoic acid; PFOS, perfluorooctane sulphonic acid; PFTrDA, perfluorotridecanoic acid; PFUnA, perfluoroundecanoic acid. Estimates show odds ratios for functional constipation per doubling of PFAS concentrations. Adjusted models included maternal age, maternal body mass index, parity, maternal smoking during pregnancy, maternal education, household income, maternal dietary habits, and Study Area as confounders. A Bonferroni correction was applied to adjust for multiple comparisons, yielding a significance threshold of P < 0.0035. PFAS concentrations were transformed using the base-2 logarithm.

### 3.3. Supplementary analyses

**Fig. 2:**
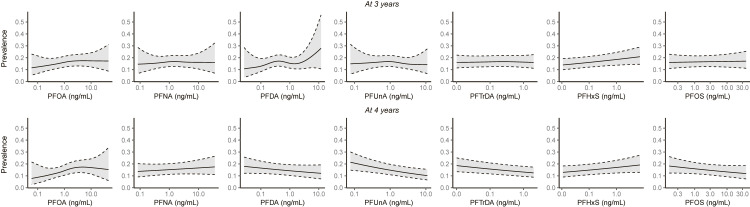
Exposure–response curves for the association between maternal PFAS concentrations and functional constipation in children. Models included maternal age, maternal body mass index, parity, maternal smoking during pregnancy, maternal education, household income, maternal dietary habits, and Study Area as confounders. PFAS, per- and polyfluoroalkyl substances; PFDA, perfluorodecanoic acid; PFHxS, perfluorohexane sulphonic acid; PFNA, perfluorononanoic acid; PFOA, perfluorooctanoic acid; PFOS, perfluorooctane sulphonic acid; PFTrDA, perfluorotridecanoic acid; PFUnA, perfluoroundecanoic acid.

A monotonic increase in odds ratios was observed for PFOA and functional constipation at 4 years when PFAS concentrations were categorized into quartiles (Supplementary Table S3). The estimated exposure–response curves were predominantly linear (Fig. 2). Multi-pollutant models yielded estimates similar to those obtained from the primary analyses by single-pollutant models, and the positive association between PFOA and functional constipation remained significant (Supplementary Table S4). The joint effect of all seven PFAS compounds showed no associations with functional constipation at either 3 or 4 years of age.

Stratification by child sex revealed no significant evidence of effect modification (Supplementary Table S5). Stratification by cesarean birth suggested that the associations of PFUnA and PFOS with functional constipation at 3 years were stronger among children delivered via cesarean delivery; however, this pattern was not observed for other PFAS compounds or for functional constipation at 4 years (Supplementary Table S6). Stratification by breastfeeding duration revealed that, when functional constipation at 3 years was the outcome, there was a positive association between PFTrDA and functional constipation among children with a breastfeeding duration of <7 months, and there was no association among those with a breastfeeding duration of ≥7 months, and the difference was statistically significant. However, this finding was not replicated for other PFAS compounds or for functional constipation at 4 years (Supplementary Table S7).

### 3.4. Sensitivity analyses

Using functional constipation at both 3 and 4 years of age as a combined outcome revealed no associations with any of the seven PFAS compounds (Supplementary Table S8). When we additionally adjusted for child sex, breastfeeding duration, and cesarean birth, the estimates were virtually unchanged from the primary analyses (Supplementary Table S9). When we additionally adjusted for daycare attendance and diaper use, both assessed at 3 and 4 years of age, the results remained largely stable (Supplementary Table S10).

Excluding variables related to maternal dietary habits yielded results consistent with the primary analysis. However, the inverse association between PFTrDA and functional constipation at 4 years reached statistical significance (P < 0.0035) (Supplementary Table S11). Excluding participants with blood samples collected at the earliest and latest 2.5% resulted in little change in the results (Supplementary Table S12). This pattern was consistent when the exclusion criteria were expanded to the earliest and latest 5% or 10% (Supplementary Tables S13 and S14).

Using modified Poisson models instead of logistic regression models produced results that were virtually unchanged from the primary analysis (Supplementary Table S15).

## 4. Discussion

Our results showed that prenatal PFOA exposure was positively associated with functional constipation in 4-year-old children in the JECS cohort. However, no associations were observed for other PFAS compounds at 4 years of age, and none of the seven PFAS compounds examined, including PFOA, were associated with functional constipation at 3 years. From a biological perspective, this isolated association between PFOA and functional constipation at 4 years seems unlikely to reflect a true causal relationship. Therefore, these results do not provide evidence for an association between prenatal PFAS exposure and functional constipation in children, but they do warrant further investigation. Similarly, although some subgroup analyses reached statistical significance, the patterns were inconsistent and sporadic, suggesting that these results are unlikely to represent true effect modification, though they nevertheless merit further study.

Epidemiological studies examining the association between PFAS exposure and functional constipation (or constipation in general) remain limited. Indeed, to our knowledge, there has been only one such study—a population-based investigation using NHANES data from U.S. adults; it found an inverse association between PFOA and constipation (odds ratio for the third vs. first tertile, 0.666; 95% CI: 0.486–0.914) [7]. Our findings differ from the previous research, likely due to substantial differences in the design of the two studies. For example, the previous study analyzed adults aged 20 years and older from the United States, while our study examined mothers and their children from Japan. Furthermore, the earlier study defined constipation based on the Bristol Stool Form Scale, while our study’s outcome was functional constipation based on the Rome III criteria. Additionally, the previous study was cross-sectional, whereas we examined prospective associations between maternal PFAS exposure and subsequent outcomes in children. Further epidemiological research will be needed to clarify the association between PFAS exposure and functional constipation.

Functional constipation in childhood is multifactorial and complex [41]. Nevertheless, several mechanisms could potentially underlie the association between prenatal PFAS exposure and childhood functional constipation. One possible mechanism involves PFAS-mediated alterations to gut microbiota, which may play a role in functional constipation pathogenesis [42]. Several epidemiological studies have reported associations of maternal PFAS levels with gut microbiota composition and its diversity in children [36, 43, 44]. However, the specific microbes implicated in these associations varied across studies. Moreover, one study reported that maternal PFAS exposure was not associated with gut microbiota outcomes in children [45]. In animal studies, PFAS exposure has been shown to alter gut microbiota in exposed mice and their offspring [46–48]. Notably, in addition to changes in gut microbiota composition, one study reported that PFOA exposure significantly decreased short-chain fatty acid levels [47], which are inversely associated with constipation severity [49].

Alternative mechanisms beyond gut microbiota modifications may also be considered. Maternal PFAS exposure may disrupt serotonin metabolism during pregnancy [50], a process linked to enteric nervous system abnormalities and increased gastrointestinal symptoms in children [51–53]. Furthermore, children are directly exposed to PFAS from the mother through transplacental transfer and breastfeeding [54, 55]. PFAS transferred from mothers to children may enhance oxidative stress [56], potentially leading to enteric nervous system dysfunction and gastrointestinal symptoms [57]. Postnatal PFAS exposure also suppresses the synthesis of bile acids, some of which function as natural laxatives [58], although this suppression has not been observed with prenatal exposure [59]. Conversely, individual PFAS exposure has been associated with elevated levels of pro-inflammatory cytokines, which may increase fecal volume and moisture content [7]. Therefore, children’s PFAS exposure through maternal transfer may confer protection against constipation.

Our findings revealed differing patterns in the associations between prenatal PFAS exposure and functional constipation at ages 3 and 4 years. As noted, we primarily interpret the single significant association with PFOA at age 4 as a chance finding. However, several potential differences between ages 3 and 4 years may alternatively explain this discrepancy. For example, previous research has shown that the gut microbiota continues to develop until age 4 [60], and differences in microbial composition between these ages may account for our observed pattern. In addition to gut microbiota, the enteric nervous system and bile acid metabolism are still developing [61–63], which may have also contributed. Moreover, normal bowel frequency is known to decrease as children grow [64]. Beyond biological differences between the two ages, differences in the social environment, including toilet training progress and daycare attendance, may have played a role [39], although additional adjustment for these variables did not alter our results. Overall, although differences exist between ages 3 and 4 years, we found no strong evidence that children are particularly vulnerable to prenatal PFAS exposure at age 4 but not at age 3.

The observed prevalence of functional constipation in this study was 11.3% at 3 years and 9.8% at 4 years, which is consistent with previous findings. A 2018 meta-analysis including children aged 0 to 18 years reported a pooled prevalence of 9.5% (95% CI: 7.5–12.1%) globally and 6.3% (95% CI: 4.0–9.6%) in Asian populations [3]. However, a more recent meta-analysis focusing on Asian children aged 0 to 18 years found a pooled prevalence of 12.0% (95% CI: 9.3–14.6%), and a prevalence of 13.4% (95% CI: 8.9–17.9%) among a subgroup of children aged 1 to 9 years [4]. The latter findings are more consistent with the current observations than those from the 2018 meta-analysis.

A key strength of this study is that it is among the first to investigate the association between maternal PFAS exposure and childhood constipation using a relatively large dataset. However, this study has several limitations. First, we defined functional constipation status through responses to self-reported questionnaires, which are subjective. We lacked information on medical diagnoses and corresponding medical treatment records. Additionally, data on maternal constipation were unavailable. Although this variable was not required for estimating the association of interest, it could inform further analysis. Second, we had no data on the gut microbiota of enrolled children and thus could not explore its potential role. Third, given the epidemiological nature of this study and the limited existing literature, definitive conclusions regarding the association of interest cannot be drawn, and alternative interpretations of the findings cannot be excluded. Finally, this is a secondary analysis of a previously collected dataset, and the analyses were not prespecified prior to data collection. Although the hypothesis examined was informed by prior research, the exploratory nature of this investigation should be acknowledged, and findings should be interpreted with appropriate caution.

## 5. Conclusions

In summary, we observed one positive association between prenatal PFOA exposure and functional constipation in children at 4 years. No associations were observed between other PFAS compounds and functional constipation at 4 years, or between any of the examined PFAS and functional constipation at 3 years. This isolated finding linking PFOA to functional constipation at 4 years is unlikely to reflect a true causal relationship given limited biological plausibility. Our results do not provide evidence of an association between prenatal PFAS exposure and functional constipation in children, though further investigation is warranted.

## Abbreviations

CI: confidence interval
JECS: Japan Environment and Children’s Study
LCMRL: lowest concentration minimum reporting level
NHANES: National Health and Nutrition Examination Survey
PFAS: per- and polyfluoroalkyl substances
PFDA: perfluorodecanoic acid
PFHxS: perfluorohexane sulphonic acid
PFNA: perfluorononanoic acid
PFOA: perfluorooctanoic acid
PFOS: perfluorooctane sulphonic acid
PFTrDA: perfluorotridecanoic acid
PFUnA: perfluoroundecanoic acid

## Supplementary information

**Additional file 1:** **Table S1.** Distribution of maternal blood PFAS concentrations (ng/mL) for all 28 compounds analyzed among participants in the Japan Environment and Children’s Study included in this analysis (n = 17,686). **Table S2.** Spearman correlation coefficients of maternal blood PFAS concentrations among participants in the Japan Environment and Children’s Study included in this analysis (n = 17,686). **Table S3.** Associations between maternal PFAS concentrations and functional constipation in children at ages 3 and 4 years in the Japan Environment and Children’s Study, analyzed by PFAS quartiles (n = 17,686). **Table S4.** Associations between maternal PFAS concentrations and functional constipation in children at ages 3 and 4 years in the Japan Environment and Children’s Study, using multi-pollutant models (n = 17,686). **Table S5.** Associations between maternal PFAS concentrations and functional constipation in children at ages 3 and 4 years in the Japan Environment and Children’s Study, stratified by child sex (n = 17,686). **Table S6.** Associations between maternal PFAS concentrations and functional constipation in children at ages 3 and 4 years in the Japan Environment and Children’s Study, stratified by cesarean birth (n = 17,646). **Table S7.** Associations between maternal PFAS concentrations and functional constipation in children at ages 3 and 4 years in the Japan Environment and Children’s Study, stratified by breastfeeding duration (n = 17,445). **Table S8.** Associations between maternal PFAS concentrations and functional constipation in children at ages 3 and 4 years in the Japan Environment and Children’s Study, using functional constipation at both 3 and 4 years of age as the outcome (n = 17,686). **Table S9.** Associations between maternal PFAS concentrations and functional constipation in children at ages 3 and 4 years in the Japan Environment and Children’s Study, with additional adjustment for child sex, cesarean birth, or breastfeeding duration (n = 17,686). **Table S10.** Associations between maternal PFAS concentrations and functional constipation in children at ages 3 and 4 years in the Japan Environment and Children’s Study, with additional adjustment for daycare attendance and diaper use during sleep (n = 17,686). **Table S11.** Associations between maternal PFAS concentrations and functional constipation in children at ages 3 and 4 years in the Japan Environment and Children’s Study, without adjustment for maternal dietary habits (n = 17,686). **Table S12.** Associations between maternal PFAS concentrations and functional constipation in children at ages 3 and 4 years in the Japan Environment and Children’s Study, excluding participants with blood samples collected at the earliest and latest 2.5% of gestational weeks (n = 16,656). **Table S13.** Associations between maternal PFAS concentrations and functional constipation in children at ages 3 and 4 years in the Japan Environment and Children’s Study, excluding participants with blood samples collected at the earliest and latest 5% of gestational weeks (n = 15,745). **Table S14.** Associations between maternal PFAS concentrations and functional constipation in children at ages 3 and 4 years in the Japan Environment and Children’s Study, excluding participants with blood samples collected at the earliest and latest 10% of gestational weeks (n = 14,066). **Table S15.** Associations between maternal PFAS concentrations and functional constipation in children at ages 3 and 4 years in the Japan Environment and Children’s Study, using modified Poisson models (n = 17,686). **Figure S1.** Directed acyclic graphs illustrating the hypothesized association between maternal PFAS exposure and child functional constipation. **Appendix S1.** DAGitty model code for the directed acyclic graphs illustrating the hypothesized association between maternal PFAS exposure and child functional constipation.

## Declarations

### Availability of data and materials

Data are unsuitable for public deposition due to ethical restrictions and legal framework of Japan. It is prohibited by the Act on the Protection of Personal Information (Act No. 57 of 30 May 2003, amendment on 9 September 2015) to publicly deposit the data containing personal information. Ethical Guidelines for Medical and Health Research Involving Human Subjects enforced by the Japan Ministry of Education, Culture, Sports, Science and Technology and the Ministry of Health, Labour and Welfare also restricts the open sharing of the epidemiologic data. All inquiries about access to data should be sent to: jecs-en@nies.go.jp. The person responsible for handling enquiries sent to this e-mail address is Dr Shoji F. Nakayama, JECS Programme Office, National Institute for Environmental Studies.
