# Supplementary material for: Associations between maternal exposure to per- and polyfluoroalkyl substances and functional constipation in children at 3 and 4 years: the Japan Environment and Children’s Study
Source: Environ Health Prev Med. 2026 May 21;31:34. doi: 10.1265/ehpm.25-00399 (PMC13222743; doi:10.1265/ehpm.25-00399)
Supplement: Supplementary file 1 — Additional file 1: Table S1. Distribution of maternal blood PFAS concentrations (ng/mL) for all 28 compounds analyzed among participants in the Japan Environment and Children’s Study included in this analysis (n = 17,686). Table S2. Spearman correlation coefficients of maternal blood PFAS concentrations among participants in the Japan Environment and Children’s Study included in this analysis (n = 17,686). Table S3. Associations between maternal PFAS concentrations and functional constipation in children at ages 3 and 4 years in the Japan Environment and Children’s Study, analyzed by PFAS quartiles (n = 17,686). Table S4. Associations between maternal PFAS concentrations and functional constipation in children at ages 3 and 4 years in the Japan Environment and Children’s Study, using multi-pollutant models (n = 17,686). Table S5. Associations between maternal PFAS concentrations and functional constipation in children at ages 3 and 4 years in the Japan Environment and Children’s Study, stratified by child sex (n = 17,686). Table S6. Associations between maternal PFAS concentrations and functional constipation in children at ages 3 and 4 years in the Japan Environment and Children’s Study, stratified by cesarean birth (n = 17,646). Table S7. Associations between maternal PFAS concentrations and functional constipation in children at ages 3 and 4 years in the Japan Environment and Children’s Study, stratified by breastfeeding duration (n = 17,445). Table S8. Associations between maternal PFAS concentrations and functional constipation in children at ages 3 and 4 years in the Japan Environment and Children’s Study, using functional constipation at both 3 and 4 years of age as the outcome (n = 17,686). Table S9. Associations between maternal PFAS concentrations and functional constipation in children at ages 3 and 4 years in the Japan Environment and Children’s Study, with additional adjustment for child sex, cesarean birth, or breastfeeding duration (n = 17,686). Table [file ehpm-31-034-s001.docx]

# Table S1. Distribution of maternal blood PFAS concentrations (ng/mL) for all 28 compounds analyzed among participants in the Japan Environment and Children’s Study included in this analysis (n=17,686)

| **Full name** | **Abbrevation** | **LCMRL** | **% exceeding LCMRL** | **Minimum** | **25th percentile** | **Median** | **75th percentile** | **Maximum** |
| --- | --- | --- | --- | --- | --- | --- | --- | --- |
| Perfluorobutanoic acid | PFBA | 0.13–0.34 | 1.45 | <LCMRL | <LCMRL | <LCMRL | <LCMRL | 1,500 |
| Perfluoropentanoic acid | PFPA | 0.13–0.27 | 0.50 | <LCMRL | <LCMRL | <LCMRL | <LCMRL | 0.91 |
| Perfluorohexanoic acid | PFHxA | 0.095–0.34 | 0.29 | <LCMRL | <LCMRL | <LCMRL | <LCMRL | 0.57 |
| Perfluoroheptanoic acid | PFHpA | 0.092–0.27 | 9.10 | <LCMRL | <LCMRL | <LCMRL | <LCMRL | 1.9 |
| Perfluorooctanoic acid | PFOA | 0.11–0.28 | 99.99 | <LCMRL | 1.1 | 1.6 | 2.5 | 45 |
| Perfluorononanoic acid | PFNA | 0.094–0.30 | 99.99 | <LCMRL | 1.0 | 1.4 | 1.9 | 36 |
| Perfluorododecanoic acid | PFDA | 0.093–0.22 | 99.53 | <LCMRL | 0.38 | 0.50 | 0.69 | 12 |
| Perfluoroundecanoic acid | PFUnA | 0.098–0.27 | 99.94 | <LCMRL | 0.85 | 1.1 | 1.5 | 11 |
| Perfluorododecanoic acid | PFDoA | 0.095–0.24 | 50.89 | <LCMRL | <LCMRL | 0.11 | 0.17 | 1.2 |
| Perfluorotridecanoic acid | PFTrDA | 0.10–0.22 | 82.16 | <LCMRL | 0.18 | 0.26 | 0.36 | 2.0 |
| Perfluorotetradecanoic acid | PFTeDA | 0.12–0.25 | 0.06 | <LCMRL | <LCMRL | <LCMRL | <LCMRL | 0.19 |
| Perfluorohexadecanoic acid | PFHxDA | 0.10–0.30 | 0.02 | <LCMRL | <LCMRL | <LCMRL | <LCMRL | 0.12 |
| Perfluorooctadecanoic acid | PFODA | 0.093–0.24 | 0.02 | <LCMRL | <LCMRL | <LCMRL | <LCMRL | 0.11 |
| Perfluorobutane sulphonic acid | PFBS | 0.080–0.21 | 0.51 | <LCMRL | <LCMRL | <LCMRL | <LCMRL | 2.8 |
| Perfluorohexane sulphonic acid | PFHxS | 0.10–0.33 | 93.23 | <LCMRL | 0.23 | 0.33 | 0.48 | 7.2 |
| Perfluoroheptane sulphonic acid | PFHpS | 0.084–0.26 | 2.94 | <LCMRL | <LCMRL | <LCMRL | <LCMRL | 0.87 |
| Perfluorooctane sulphonic acid | PFOS | 0.096–0.32 | 99.99 | <LCMRL | 2.1 | 3.0 | 4.1 | 39 |
| Perfluorodecane sulphonic acid | PFDS | 0.11–0.27 | 0.00 | <LCMRL | <LCMRL | <LCMRL | <LCMRL | <LCMRL |
| N-Methyl perfluorooctane sulphonamido acetic acid | MeFOSA-A | 0.088–0.26 | 1.04 | <LCMRL | <LCMRL | <LCMRL | <LCMRL | 2.5 |
| N-Ethyl perfluorooctane sulphonamido acetic acid | EtFOSA-A | 0.11–0.29 | 0.02 | <LCMRL | <LCMRL | <LCMRL | <LCMRL | 0.42 |
| N-Methyl perfluorooctane sulphonamide | MeFOSA-M | 0.11–0.28 | 0.06 | <LCMRL | <LCMRL | <LCMRL | <LCMRL | 0.70 |
| N-Ethyl perfluorooctane sulphonamide | EtFOSA-M | 0.099–0.28 | 0.00 | <LCMRL | <LCMRL | <LCMRL | <LCMRL | <LCMRL |
| 4:2 Fluorotelomer sulphonic acid | 4:2FTS | 0.082–0.24 | 0.00 | <LCMRL | <LCMRL | <LCMRL | <LCMRL | <LCMRL |
| 6:2 Fluorotelomer sulphonic acid | 6:2FTS | 0.080–0.32 | 0.03 | <LCMRL | <LCMRL | <LCMRL | <LCMRL | 0.27 |
| 8:2 Fluorotelomer sulphonic acid | 8:2FTS | 0.10–0.23 | 0.01 | <LCMRL | <LCMRL | <LCMRL | <LCMRL | 0.13 |
| 6:2 Polyfluoroalkyl phosphoric acid diester | 6:2diPAP | 0.10–0.27 | 2.23 | <LCMRL | <LCMRL | <LCMRL | <LCMRL | 1.8 |
| 8:2 Polyfluoroalkyl phosphoric acid diester | 8:2diPAP | 0.096–0.23 | 0.18 | <LCMRL | <LCMRL | <LCMRL | <LCMRL | 0.86 |
| Sodium bis-[2-(N-ethylperfluorooctane-1-sulfonamido)ethyl] phosphate | diSAmPAP | 0.11–0.27 | 0.00 | <LCMRL | <LCMRL | <LCMRL | <LCMRL | <LCMRL |

Note: LCMRL, lowest concentration minimum reporting level.

# Table S2. Spearman correlation coefficients of maternal blood PFAS concentrations among participants in the Japan Environment and Children’s Study included in this analysis (n=17,686)

| **Value** | **PFOA** | **PFNA** | **PFDA** | **PFUnA** | **PFTrDA** | **PFHxS** | **PFOS** |
| --- | --- | --- | --- | --- | --- | --- | --- |
| PFOA | 1.00 | 0.74 | 0.61 | 0.27 | 0.17 | 0.60 | 0.55 |
| PFNA |  | 1.00 | 0.73 | 0.48 | 0.33 | 0.49 | 0.65 |
| PFDA |  |  | 1.00 | 0.83 | 0.64 | 0.36 | 0.80 |
| PFUnA |  |  |  | 1.00 | 0.85 | 0.19 | 0.71 |
| PFTrDA |  |  |  |  | 1.00 | 0.10 | 0.54 |
| PFHxS |  |  |  |  |  | 1.00 | 0.56 |
| PFOS |  |  |  |  |  |  | 1.00 |

Note: PFAS, per- and polyfluoroalkyl substances; PFDA, perfluorodecanoic acid; PFHxS, perfluorohexane sulphonic acid; PFNA, perfluorononanoic acid; PFOA, perfluorooctanoic acid; PFOS, perfluorooctane sulphonic acid; PFTrDA, perfluorotridecanoic acid; PFUnA, perfluoroundecanoic acid.

# Table S3. Associations between maternal PFAS concentrations and functional constipation in children at ages 3 and 4 years in the Japan Environment and Children’s Study, analyzed by PFAS quartiles (n=17,686)

|  |  | **1st quartile** | **2nd quartile** | **3rd quartile** | **4th quartile** |  |
| --- | --- | --- | --- | --- | --- | --- |
| **Outcome assessment** | **PFAS** | **—** | **Odds ratio (95% CI)** | **Odds ratio (95% CI)** | **Odds ratio (95% CI)** | **p for trend** |
| At 3 years | PFOA | Ref. | 1.15 (1.00–1.33) | 1.17 (1.02–1.35) | 1.16 (0.99–1.36) | 0.0743 |
|  | PFNA | Ref. | 1.07 (0.93–1.22) | 1.04 (0.90–1.20) | 1.01 (0.87–1.18) | 0.9829 |
|  | PFDA | Ref. | 1.00 (0.87–1.14) | 1.01 (0.89–1.16) | 0.91 (0.78–1.06) | 0.3075 |
|  | PFUnA | Ref. | 1.02 (0.88–1.18) | 1.01 (0.89–1.15) | 0.91 (0.79–1.05) | 0.2503 |
|  | PFTrDA | Ref. | 1.07 (0.93–1.22) | 1.11 (0.97–1.27) | 1.01 (0.88–1.17) | 0.6917 |
|  | PFHxS | Ref. | 1.05 (0.91–1.21) | 1.06 (0.91–1.22) | 1.15 (0.98–1.35) | 0.1097 |
|  | PFOS | Ref. | 1.06 (0.92–1.21) | 1.13 (0.98–1.31) | 1.00 (0.85–1.18) | 0.8120 |
| At 4 years | PFOA | Ref. | 1.29 (1.11–1.51) | 1.36 (1.16–1.59) | 1.41 (1.19–1.68) | 0.0001 |
|  | PFNA | Ref. | 1.00 (0.86–1.16) | 0.95 (0.82–1.11) | 1.10 (0.94–1.29) | 0.2994 |
|  | PFDA | Ref. | 1.06 (0.92–1.22) | 0.95 (0.82–1.10) | 0.90 (0.77–1.06) | 0.1196 |
|  | PFUnA | Ref. | 0.93 (0.80–1.09) | 0.90 (0.78–1.03) | 0.79 (0.68–0.92) | 0.0031 |
|  | PFTrDA | Ref. | 0.93 (0.81–1.07) | 0.93 (0.81–1.08) | 0.79 (0.68–0.93) | 0.0074 |
|  | PFHxS | Ref. | 1.06 (0.91–1.23) | 1.05 (0.90–1.23) | 1.18 (1.00–1.40) | 0.0778 |
|  | PFOS | Ref. | 1.04 (0.90–1.20) | 0.93 (0.80–1.09) | 0.87 (0.73–1.03) | 0.0425 |

Note: PFAS, per- and polyfluoroalkyl substances; PFDA, perfluorodecanoic acid; PFHxS, perfluorohexane sulphonic acid; PFNA, perfluorononanoic acid; PFOA, perfluorooctanoic acid; PFOS, perfluorooctane sulphonic acid; PFTrDA, perfluorotridecanoic acid; PFUnA, perfluoroundecanoic acid. Estimates show odds ratios for functional constipation in each quartile relative to the first quartile (reference). Models included maternal age, maternal body mass index, parity, maternal smoking during pregnancy, maternal education, household income, maternal dietary habits, and Study Area as confounders.

# Table S4. Associations between maternal PFAS concentrations and functional constipation in children at ages 3 and 4 years in the Japan Environment and Children’s Study, using multi-pollutant models (n=17,686)

|  |  | **Crude** |  | **Adjusted** |  |
| --- | --- | --- | --- | --- | --- |
| **Outcome assessment** | **PFAS** | **Odds ratio (95% CI)** | **p** | **Odds ratio (95% CI)** | **p** |
| At 3 years | PFOA | 1.11 (1.02–1.21) | 0.0190 | 1.07 (0.97–1.19) | 0.1605 |
|  | PFNA | 0.92 (0.81–1.03) | 0.1553 | 0.94 (0.83–1.06) | 0.3235 |
|  | PFDA | 0.97 (0.82–1.15) | 0.7593 | 1.03 (0.86–1.23) | 0.7750 |
|  | PFUnA | 0.89 (0.75–1.06) | 0.2024 | 0.86 (0.71–1.04) | 0.1106 |
|  | PFTrDA | 1.04 (0.98–1.11) | 0.1718 | 1.05 (0.99–1.11) | 0.1381 |
|  | PFHxS | 1.04 (0.98–1.11) | 0.1560 | 1.04 (0.98–1.11) | 0.2249 |
|  | PFOS | 0.98 (0.86–1.13) | 0.8189 | 1.03 (0.89–1.20) | 0.6855 |
|  | Joint effect | 0.95 (0.87–1.03) | 0.2084 | 1.00 (0.90–1.10) | 0.9371 |
| At 4 years | PFOA | 1.22 (1.11–1.34) | <0.0001 | 1.21 (1.08–1.34) | 0.0006 |
|  | PFNA | 0.99 (0.87–1.12) | 0.8392 | 0.99 (0.86–1.12) | 0.8286 |
|  | PFDA | 0.88 (0.73–1.05) | 0.1525 | 0.93 (0.77–1.12) | 0.4244 |
|  | PFUnA | 1.08 (0.89–1.30) | 0.4228 | 0.98 (0.80–1.20) | 0.8789 |
|  | PFTrDA | 0.95 (0.90–1.01) | 0.0982 | 0.96 (0.91–1.02) | 0.2433 |
|  | PFHxS | 1.04 (0.98–1.11) | 0.2216 | 1.04 (0.97–1.11) | 0.3179 |
|  | PFOS | 0.88 (0.76–1.01) | 0.0693 | 0.92 (0.79–1.08) | 0.3264 |
|  | Join effect | 0.99 (0.90–1.08) | 0.7505 | 1.00 (0.90–1.11) | 0.9832 |

Note: PFAS, per- and polyfluoroalkyl substances; PFDA, perfluorodecanoic acid; PFHxS, perfluorohexane sulphonic acid; PFNA, perfluorononanoic acid; PFOA, perfluorooctanoic acid; PFOS, perfluorooctane sulphonic acid; PFTrDA, perfluorotridecanoic acid; PFUnA, perfluoroundecanoic acid. Estimates show odds ratios for functional constipation per doubling of PFAS concentrations. Adjusted models included maternal age, maternal body mass index, parity, maternal smoking during pregnancy, maternal education, household income, maternal dietary habits, and Study Area as confounders. A Bonferroni correction was applied to adjust for multiple comparisons, yielding a significance threshold of P < 0.0035. PFAS concentrations were transformed using the base-2 logarithm.

# Table S5. Associations between maternal PFAS concentrations and functional constipation in children at ages 3 and 4 years in the Japan Environment and Children’s Study, stratified by child sex (n=17,686)

|  |  | **Male** | **Female** |  |
| --- | --- | --- | --- | --- |
| **Outcome assessment** | **PFAS** | **Odds ratio (95% CI)** | **Odds ratio (95% CI)** | **p for difference** |
| At 3 years | PFOA | 1.05 (0.96–1.15) | 1.07 (0.98–1.16) | 0.8415 |
|  | PFNA | 0.97 (0.87–1.09) | 1.04 (0.94–1.15) | 0.4252 |
|  | PFDA | 0.96 (0.86–1.07) | 1.04 (0.93–1.15) | 0.3078 |
|  | PFUnA | 0.90 (0.80–1.01) | 1.03 (0.92–1.15) | 0.1017 |
|  | PFTrDA | 1.00 (0.94–1.06) | 1.01 (0.96–1.07) | 0.7518 |
|  | PFHxS | 1.04 (0.96–1.12) | 1.07 (0.99–1.14) | 0.6479 |
|  | PFOS | 0.97 (0.87–1.09) | 1.05 (0.94–1.18) | 0.3285 |
| At 4 years | PFOA | 1.07 (0.98–1.18) | 1.20 (1.08–1.32) | 0.1221 |
|  | PFNA | 1.03 (0.92–1.15) | 1.04 (0.93–1.17) | 0.9161 |
|  | PFDA | 0.92 (0.83–1.03) | 0.98 (0.87–1.11) | 0.4496 |
|  | PFUnA | 0.85 (0.75–0.95) | 0.94 (0.83–1.07) | 0.2314 |
|  | PFTrDA | 0.93 (0.88–0.98) | 0.96 (0.90–1.02) | 0.4684 |
|  | PFHxS | 1.10 (1.01–1.19) | 1.01 (0.93–1.09) | 0.1270 |
|  | PFOS | 0.94 (0.84–1.06) | 0.94 (0.83–1.06) | 0.9477 |

Note: PFAS, per- and polyfluoroalkyl substances; PFDA, perfluorodecanoic acid; PFHxS, perfluorohexane sulphonic acid; PFNA, perfluorononanoic acid; PFOA, perfluorooctanoic acid; PFOS, perfluorooctane sulphonic acid; PFTrDA, perfluorotridecanoic acid; PFUnA, perfluoroundecanoic acid. Estimates show odds ratios for functional constipation per doubling of PFAS concentrations. Adjusted models included maternal age, maternal body mass index, parity, maternal smoking during pregnancy, maternal education, household income, maternal dietary habits, and Study Area as confounders. PFAS concentrations were transformed using the base-2 logarithm.

# Table S6. Associations between maternal PFAS concentrations and functional constipation in children at ages 3 and 4 years in the Japan Environment and Children’s Study, stratified by cesarean birth (n=17,646)

|  |  | **Non-cesarean birth** | **Cesarean birth** |  |
| --- | --- | --- | --- | --- |
| **Outcome assessment** | **PFAS** | **Odds ratio (95% CI)** | **Odds ratio (95% CI)** | **p for difference** |
| At 3 years | PFOA | 1.07 (0.99–1.15) | 1.01 (0.87–1.17) | 0.4862 |
|  | PFNA | 0.99 (0.91–1.08) | 1.05 (0.88–1.24) | 0.5924 |
|  | PFDA | 0.96 (0.88–1.04) | 1.16 (0.97–1.39) | 0.0584 |
|  | PFUnA | 0.91 (0.83–1.00) | 1.22 (1.02–1.48) | 0.0051 |
|  | PFTrDA | 1.00 (0.95–1.05) | 1.04 (0.94–1.15) | 0.4582 |
|  | PFHxS | 1.04 (0.98–1.10) | 1.09 (0.97–1.24) | 0.4772 |
|  | PFOS | 0.96 (0.87–1.05) | 1.28 (1.06–1.54) | 0.0060 |
| At 4 years | PFOA | 1.14 (1.06–1.23) | 1.07 (0.91–1.24) | 0.4377 |
|  | PFNA | 1.04 (0.95–1.14) | 1.01 (0.84–1.21) | 0.7820 |
|  | PFDA | 0.96 (0.88–1.05) | 0.90 (0.75–1.08) | 0.5444 |
|  | PFUnA | 0.89 (0.81–0.98) | 0.87 (0.72–1.05) | 0.8008 |
|  | PFTrDA | 0.95 (0.91–1.00) | 0.89 (0.81–0.98) | 0.2042 |
|  | PFHxS | 1.03 (0.97–1.10) | 1.14 (1.00–1.30) | 0.1580 |
|  | PFOS | 0.92 (0.84–1.02) | 1.01 (0.83–1.22) | 0.4177 |

Note: PFAS, per- and polyfluoroalkyl substances; PFDA, perfluorodecanoic acid; PFHxS, perfluorohexane sulphonic acid; PFNA, perfluorononanoic acid; PFOA, perfluorooctanoic acid; PFOS, perfluorooctane sulphonic acid; PFTrDA, perfluorotridecanoic acid; PFUnA, perfluoroundecanoic acid. Estimates show odds ratios for functional constipation per doubling of PFAS concentrations. Adjusted models included maternal age, maternal body mass index, parity, maternal smoking during pregnancy, maternal education, household income, maternal dietary habits, and Study Area as confounders. PFAS concentrations were transformed using the base-2 logarithm.

# Table S7. Associations between maternal PFAS concentrations and functional constipation in children at ages 3 and 4 years in the Japan Environment and Children’s Study, stratified by breastfeeding duration (n=17,445)

|  |  | **Breastfeeding duration <7 months** | **Breastfeeding duration ≥7 months** |  |
| --- | --- | --- | --- | --- |
| **Outcome assessment** | **PFAS** | **Odds ratio (95% CI)** | **Odds ratio (95% CI)** | **p for difference** |
| At 3 years | PFOA | 1.11 (0.97–1.26) | 1.02 (0.95–1.10) | 0.2879 |
|  | PFNA | 1.08 (0.93–1.25) | 0.98 (0.89–1.07) | 0.2554 |
|  | PFDA | 1.08 (0.93–1.25) | 0.96 (0.88–1.05) | 0.1903 |
|  | PFUnA | 1.02 (0.87–1.19) | 0.94 (0.86–1.04) | 0.4330 |
|  | PFTrDA | 1.09 (1.01–1.18) | 0.98 (0.93–1.03) | 0.0238 |
|  | PFHxS | 1.08 (0.97–1.21) | 1.04 (0.98–1.10) | 0.5026 |
|  | PFOS | 1.10 (0.94–1.28) | 0.97 (0.88–1.06) | 0.1857 |
| At 4 years | PFOA | 1.16 (1.02–1.33) | 1.09 (1.00–1.18) | 0.4046 |
|  | PFNA | 1.10 (0.94–1.29) | 1.00 (0.91–1.10) | 0.3272 |
|  | PFDA | 1.07 (0.91–1.25) | 0.91 (0.83–1.00) | 0.0927 |
|  | PFUnA | 0.98 (0.83–1.15) | 0.86 (0.78–0.95) | 0.2075 |
|  | PFTrDA | 0.98 (0.90–1.06) | 0.93 (0.89–0.98) | 0.3459 |
|  | PFHxS | 1.02 (0.91–1.14) | 1.05 (0.98–1.12) | 0.7159 |
|  | PFOS | 1.02 (0.86–1.20) | 0.90 (0.82–1.00) | 0.2292 |

Note: PFAS, per- and polyfluoroalkyl substances; PFDA, perfluorodecanoic acid; PFHxS, perfluorohexane sulphonic acid; PFNA, perfluorononanoic acid; PFOA, perfluorooctanoic acid; PFOS, perfluorooctane sulphonic acid; PFTrDA, perfluorotridecanoic acid; PFUnA, perfluoroundecanoic acid. Estimates show odds ratios for functional constipation per doubling of PFAS concentrations. Adjusted models included maternal age, maternal body mass index, parity, maternal smoking during pregnancy, maternal education, household income, maternal dietary habits, and Study Area as confounders. PFAS concentrations were transformed using the base-2 logarithm.

# Table S8. Associations between maternal PFAS concentrations and functional constipation in children at ages 3 and 4 years in the Japan Environment and Children’s Study, using functional constipation at both 3 and 4 years of age as the outcome (n=17,686)

|  | **Crude** |  | **Adjusted** |  |
| --- | --- | --- | --- | --- |
| **PFAS** | **Odds ratio (95% CI)** | **p** | **Odds ratio (95% CI)** | **p** |
| PFOA | 1.06 (0.98–1.15) | 0.1216 | 1.11 (1.00–1.23) | 0.0493 |
| PFNA | 0.95 (0.85–1.06) | 0.3369 | 0.96 (0.85–1.08) | 0.4698 |
| PFDA | 0.88 (0.79–0.97) | 0.0135 | 0.90 (0.80–1.01) | 0.0844 |
| PFUnA | 0.86 (0.77–0.96) | 0.0091 | 0.88 (0.77–1.00) | 0.0436 |
| PFTrDA | 0.96 (0.90–1.02) | 0.1648 | 0.98 (0.91–1.04) | 0.4437 |
| PFHxS | 1.04 (0.97–1.12) | 0.2722 | 1.06 (0.97–1.15) | 0.1792 |
| PFOS | 0.89 (0.80–0.99) | 0.0271 | 0.90 (0.79–1.02) | 0.0987 |

Note: PFAS, per- and polyfluoroalkyl substances; PFDA, perfluorodecanoic acid; PFHxS, perfluorohexane sulphonic acid; PFNA, perfluorononanoic acid; PFOA, perfluorooctanoic acid; PFOS, perfluorooctane sulphonic acid; PFTrDA, perfluorotridecanoic acid; PFUnA, perfluoroundecanoic acid. Estimates show odds ratios for functional constipation per doubling of PFAS concentrations. Adjusted models included maternal age, maternal body mass index, parity, maternal smoking during pregnancy, maternal education, household income, maternal dietary habits, and Study Area as confounders. PFAS concentrations were transformed using the base-2 logarithm.

# Table S9. Associations between maternal PFAS concentrations and functional constipation in children at ages 3 and 4 years in the Japan Environment and Children’s Study, with additional adjustment for child sex, cesarean birth, or breastfeeding duration (n=17,686)

|  |  | **Additionally adjusted for child sex** |  | **Additionally adjusted for cesarean birth** |  | **Additionally adjusted for breastfeeding duration** |  |
| --- | --- | --- | --- | --- | --- | --- | --- |
| **Outcome assessment** | **PFAS** | **Odds ratio (95% CI)** | **p** | **Odds ratio (95% CI)** | **p** | **Odds ratio (95% CI)** | **p** |
| At 3 years | PFOA | 1.06 (0.99–1.13) | 0.0844 | 1.06 (0.99–1.13) | 0.0855 | 1.04 (0.98–1.11) | 0.1929 |
|  | PFNA | 1.01 (0.93–1.08) | 0.8835 | 1.00 (0.93–1.08) | 0.9160 | 1.00 (0.93–1.08) | 0.9789 |
|  | PFDA | 1.00 (0.92–1.07) | 0.8995 | 0.99 (0.92–1.07) | 0.8688 | 1.00 (0.92–1.07) | 0.9346 |
|  | PFUnA | 0.96 (0.89–1.04) | 0.3320 | 0.96 (0.89–1.04) | 0.3324 | 0.97 (0.89–1.05) | 0.4495 |
|  | PFTrDA | 1.01 (0.97–1.05) | 0.7638 | 1.01 (0.97–1.05) | 0.7501 | 1.01 (0.97–1.05) | 0.6075 |
|  | PFHxS | 1.05 (1.00–1.11) | 0.0553 | 1.05 (1.00–1.11) | 0.0518 | 1.05 (0.99–1.10) | 0.0921 |
|  | PFOS | 1.01 (0.93–1.10) | 0.7877 | 1.01 (0.93–1.09) | 0.8217 | 1.01 (0.93–1.09) | 0.8472 |
| At 4 years | PFOA | 1.13 (1.05–1.21) | 0.0006 | 1.13 (1.05–1.20) | 0.0007 | 1.11 (1.04–1.19) | 0.0027 |
|  | PFNA | 1.03 (0.95–1.12) | 0.4146 | 1.03 (0.95–1.12) | 0.4308 | 1.03 (0.95–1.11) | 0.4886 |
|  | PFDA | 0.95 (0.88–1.03) | 0.2049 | 0.95 (0.88–1.03) | 0.2037 | 0.95 (0.88–1.03) | 0.2250 |
|  | PFUnA | 0.89 (0.81–0.96) | 0.0054 | 0.89 (0.81–0.97) | 0.0056 | 0.89 (0.82–0.97) | 0.0092 |
|  | PFTrDA | 0.94 (0.90–0.98) | 0.0048 | 0.94 (0.90–0.98) | 0.0048 | 0.94 (0.90–0.99) | 0.0080 |
|  | PFHxS | 1.05 (0.99–1.11) | 0.0792 | 1.05 (0.99–1.11) | 0.0866 | 1.04 (0.99–1.10) | 0.1385 |
|  | PFOS | 0.94 (0.86–1.02) | 0.1395 | 0.94 (0.86–1.02) | 0.1429 | 0.94 (0.86–1.02) | 0.1285 |

Note: PFAS, per- and polyfluoroalkyl substances; PFDA, perfluorodecanoic acid; PFHxS, perfluorohexane sulphonic acid; PFNA, perfluorononanoic acid; PFOA, perfluorooctanoic acid; PFOS, perfluorooctane sulphonic acid; PFTrDA, perfluorotridecanoic acid; PFUnA, perfluoroundecanoic acid. Estimates show odds ratios for functional constipation per doubling of PFAS concentrations. Models included maternal age, maternal body mass index, parity, maternal smoking during pregnancy, maternal education, household income, maternal dietary habits, and Study Area as confounders. Each model was further adjusted for child sex, cesarean birth, or breastfeeding duration as a confounder. A Bonferroni correction was applied to adjust for multiple comparisons, yielding a significance threshold of P < 0.0035. PFAS concentrations were transformed using the base-2 logarithm.

# Table S10. Associations between maternal PFAS concentrations and functional constipation in children at ages 3 and 4 years in the Japan Environment and Children’s Study, with additional adjustment for daycare attendance and diaper use during sleep (n=17,686)

|  |  | **Adjusted** |  |
| --- | --- | --- | --- |
| **Outcome assessment** | **PFAS** | **Odds ratio (95% CI)** | **p** |
| At 3 years | PFOA | 1.06 (0.99–1.13) | 0.0763 |
|  | PFNA | 1.01 (0.93–1.09) | 0.8431 |
|  | PFDA | 1.00 (0.93–1.08) | 0.9839 |
|  | PFUnA | 0.96 (0.89–1.05) | 0.3857 |
|  | PFTrDA | 1.01 (0.97–1.05) | 0.6897 |
|  | PFHxS | 1.05 (1.00–1.11) | 0.0518 |
|  | PFOS | 1.02 (0.94–1.10) | 0.7060 |
| At 4 years | PFOA | 1.12 (1.05–1.20) | 0.0008 |
|  | PFNA | 1.03 (0.95–1.12) | 0.4133 |
|  | PFDA | 0.95 (0.88–1.03) | 0.2260 |
|  | PFUnA | 0.89 (0.82–0.97) | 0.0080 |
|  | PFTrDA | 0.94 (0.90–0.99) | 0.0081 |
|  | PFHxS | 1.05 (0.99–1.11) | 0.0817 |
|  | PFOS | 0.94 (0.87–1.03) | 0.1800 |

Note: PFAS, per- and polyfluoroalkyl substances; PFDA, perfluorodecanoic acid; PFHxS, perfluorohexane sulphonic acid; PFNA, perfluorononanoic acid; PFOA, perfluorooctanoic acid; PFOS, perfluorooctane sulphonic acid; PFTrDA, perfluorotridecanoic acid; PFUnA, perfluoroundecanoic acid. Estimates show odds ratios for functional constipation per doubling of PFAS concentrations. Models included maternal age, maternal body mass index, parity, maternal smoking during pregnancy, maternal education, household income, Study Area, daycare attendance, and diaper use during sleep as confounders. Daycare attendance and diaper use were assessed at the same time point as the outcome assessment. A Bonferroni correction was applied to adjust for multiple comparisons, yielding a significance threshold of P < 0.0035. PFAS concentrations were transformed using the base-2 logarithm.

# Table S11. Associations between maternal PFAS concentrations and functional constipation in children at ages 3 and 4 years in the Japan Environment and Children’s Study, without adjustment for maternal dietary habits (n=17,686)

|  |  | **Adjusted** |  |
| --- | --- | --- | --- |
| **Outcome assessment** | **PFAS** | **Odds ratio (95% CI)** | **p** |
| At 3 years | PFOA | 1.05 (0.99–1.12) | 0.1205 |
|  | PFNA | 1.00 (0.93–1.08) | 0.9925 |
|  | PFDA | 0.99 (0.92–1.06) | 0.7252 |
|  | PFUnA | 0.96 (0.88–1.03) | 0.2487 |
|  | PFTrDA | 1.00 (0.96–1.05) | 0.8581 |
|  | PFHxS | 1.05 (1.00–1.11) | 0.0442 |
|  | PFOS | 1.00 (0.93–1.09) | 0.9193 |
| At 4 years | PFOA | 1.12 (1.05–1.20) | 0.0009 |
|  | PFNA | 1.03 (0.95–1.12) | 0.4651 |
|  | PFDA | 0.95 (0.87–1.02) | 0.1648 |
|  | PFUnA | 0.89 (0.81–0.96) | 0.0040 |
|  | PFTrDA | 0.94 (0.90–0.98) | 0.0033 |
|  | PFHxS | 1.05 (1.00–1.11) | 0.0716 |
|  | PFOS | 0.94 (0.86–1.02) | 0.1253 |

Note: PFAS, per- and polyfluoroalkyl substances; PFDA, perfluorodecanoic acid; PFHxS, perfluorohexane sulphonic acid; PFNA, perfluorononanoic acid; PFOA, perfluorooctanoic acid; PFOS, perfluorooctane sulphonic acid; PFTrDA, perfluorotridecanoic acid; PFUnA, perfluoroundecanoic acid. Estimates show odds ratios for functional constipation per doubling of PFAS concentrations. Models included maternal age, maternal body mass index, parity, maternal smoking during pregnancy, maternal education, household income, and Study Area as confounders. A Bonferroni correction was applied to adjust for multiple comparisons, yielding a significance threshold of P < 0.0035. PFAS concentrations were transformed using the base-2 logarithm.

# Table S12. Associations between maternal PFAS concentrations and functional constipation in children at ages 3 and 4 years in the Japan Environment and Children’s Study, excluding participants with blood samples collected at the earliest and latest 2.5% of gestational weeks (n = 16,656)

|  |  | **Crude** |  | **Adjusted** |  |
| --- | --- | --- | --- | --- | --- |
| **Outcome assessment** | **PFAS** | **Risk ratio (95% CI)** | **p** | **Risk ratio (95% CI)** | **p** |
| At 3 years | PFOA | 1.05 (1.00–1.10) | 0.0632 | 1.06 (0.99–1.13) | 0.1084 |
|  | PFNA | 0.98 (0.92–1.05) | 0.6541 | 1.00 (0.93–1.08) | 0.9525 |
|  | PFDA | 0.96 (0.90–1.03) | 0.2243 | 0.99 (0.91–1.07) | 0.7469 |
|  | PFUnA | 0.93 (0.86–1.00) | 0.0477 | 0.96 (0.88–1.04) | 0.2928 |
|  | PFTrDA | 0.99 (0.95–1.03) | 0.5753 | 1.00 (0.96–1.05) | 0.8368 |
|  | PFHxS | 1.05 (1.00–1.10) | 0.0446 | 1.06 (1.00–1.11) | 0.0465 |
|  | PFOS | 0.98 (0.91–1.04) | 0.4920 | 1.01 (0.93–1.09) | 0.8938 |
| At 4 years | PFOA | 1.11 (1.05–1.17) | 0.0002 | 1.13 (1.06–1.22) | 0.0004 |
|  | PFNA | 1.03 (0.96–1.11) | 0.4167 | 1.03 (0.95–1.12) | 0.4613 |
|  | PFDA | 0.93 (0.87–1.00) | 0.0621 | 0.94 (0.86–1.02) | 0.1260 |
|  | PFUnA | 0.88 (0.81–0.95) | 0.0010 | 0.88 (0.80–0.96) | 0.0040 |
|  | PFTrDA | 0.93 (0.90–0.97) | 0.0010 | 0.94 (0.90–0.99) | 0.0090 |
|  | PFHxS | 1.05 (1.00–1.10) | 0.0548 | 1.05 (0.99–1.11) | 0.1138 |
|  | PFOS | 0.93 (0.87–1.00) | 0.0521 | 0.93 (0.85–1.01) | 0.0851 |

Note: PFAS, per- and polyfluoroalkyl substances; PFDA, perfluorodecanoic acid; PFHxS, perfluorohexane sulphonic acid; PFNA, perfluorononanoic acid; PFOA, perfluorooctanoic acid; PFOS, perfluorooctane sulphonic acid; PFTrDA, perfluorotridecanoic acid; PFUnA, perfluoroundecanoic acid. Estimates show odds ratios for functional constipation per doubling of PFAS concentrations. Models included maternal age, maternal body mass index, parity, maternal smoking during pregnancy, maternal education, household income, and Study Area as confounders. A Bonferroni correction was applied to adjust for multiple comparisons, yielding a significance threshold of P < 0.0035. PFAS concentrations were transformed using the base-2 logarithm.

# Table S13. Associations between maternal PFAS concentrations and functional constipation in children at ages 3 and 4 years in the Japan Environment and Children’s Study, excluding participants with blood samples collected at the earliest and latest 5% of gestational weeks (n = 15,745)

|  |  | **Crude** |  | **Adjusted** |  |
| --- | --- | --- | --- | --- | --- |
| **Outcome assessment** | **PFAS** | **Risk ratio (95% CI)** | **p** | **Risk ratio (95% CI)** | **p** |
| At 3 years | PFOA | 1.06 (1.01–1.12) | 0.0234 | 1.07 (1.00–1.15) | 0.0383 |
|  | PFNA | 1.00 (0.93–1.07) | 0.9629 | 1.02 (0.94–1.10) | 0.7085 |
|  | PFDA | 0.97 (0.90–1.04) | 0.3345 | 1.00 (0.92–1.08) | 0.9436 |
|  | PFUnA | 0.93 (0.86–1.00) | 0.0560 | 0.96 (0.88–1.05) | 0.3707 |
|  | PFTrDA | 0.99 (0.95–1.03) | 0.5065 | 1.00 (0.96–1.05) | 0.8804 |
|  | PFHxS | 1.06 (1.01–1.11) | 0.0276 | 1.06 (1.00–1.12) | 0.0390 |
|  | PFOS | 0.99 (0.92–1.06) | 0.8036 | 1.02 (0.94–1.11) | 0.6016 |
| At 4 years | PFOA | 1.12 (1.06–1.18) | <0.0001 | 1.14 (1.06–1.22) | 0.0004 |
|  | PFNA | 1.04 (0.96–1.12) | 0.3475 | 1.03 (0.95–1.13) | 0.4373 |
|  | PFDA | 0.94 (0.87–1.01) | 0.0791 | 0.93 (0.86–1.02) | 0.1219 |
|  | PFUnA | 0.88 (0.81–0.95) | 0.0014 | 0.88 (0.80–0.96) | 0.0052 |
|  | PFTrDA | 0.93 (0.89–0.97) | 0.0009 | 0.94 (0.90–0.98) | 0.0081 |
|  | PFHxS | 1.05 (1.00–1.10) | 0.0643 | 1.04 (0.98–1.10) | 0.2155 |
|  | PFOS | 0.94 (0.87–1.01) | 0.1064 | 0.93 (0.85–1.02) | 0.1267 |

Note: PFAS, per- and polyfluoroalkyl substances; PFDA, perfluorodecanoic acid; PFHxS, perfluorohexane sulphonic acid; PFNA, perfluorononanoic acid; PFOA, perfluorooctanoic acid; PFOS, perfluorooctane sulphonic acid; PFTrDA, perfluorotridecanoic acid; PFUnA, perfluoroundecanoic acid. Estimates show odds ratios for functional constipation per doubling of PFAS concentrations. Models included maternal age, maternal body mass index, parity, maternal smoking during pregnancy, maternal education, household income, and Study Area as confounders. A Bonferroni correction was applied to adjust for multiple comparisons, yielding a significance threshold of P < 0.0035. PFAS concentrations were transformed using the base-2 logarithm.

# Table S14. Associations between maternal PFAS concentrations and functional constipation in children at ages 3 and 4 years in the Japan Environment and Children’s Study, excluding participants with blood samples collected at the earliest and latest 10% of gestational weeks (n = 14,066)

|  |  | **Crude** |  | **Adjusted** |  |
| --- | --- | --- | --- | --- | --- |
| **Outcome assessment** | **PFAS** | **Risk ratio (95% CI)** | **p** | **Risk ratio (95% CI)** | **p** |
| At 3 years | PFOA | 1.05 (0.99–1.11) | 0.0811 | 1.07 (0.99–1.15) | 0.0700 |
|  | PFNA | 0.99 (0.92–1.06) | 0.7289 | 1.01 (0.93–1.10) | 0.8188 |
|  | PFDA | 0.95 (0.89–1.02) | 0.1897 | 0.99 (0.91–1.08) | 0.8155 |
|  | PFUnA | 0.92 (0.85–1.00) | 0.0392 | 0.95 (0.87–1.04) | 0.3039 |
|  | PFTrDA | 0.97 (0.93–1.02) | 0.2201 | 0.99 (0.95–1.04) | 0.6966 |
|  | PFHxS | 1.04 (0.99–1.10) | 0.1026 | 1.05 (0.99–1.11) | 0.0953 |
|  | PFOS | 0.97 (0.90–1.04) | 0.4095 | 1.01 (0.92–1.10) | 0.8719 |
| At 4 years | PFOA | 1.11 (1.04–1.17) | 0.0009 | 1.13 (1.05–1.22) | 0.0011 |
|  | PFNA | 1.02 (0.95–1.11) | 0.5577 | 1.03 (0.94–1.13) | 0.4926 |
|  | PFDA | 0.92 (0.86–1.00) | 0.0423 | 0.93 (0.85–1.02) | 0.1095 |
|  | PFUnA | 0.86 (0.79–0.94) | 0.0007 | 0.87 (0.79–0.95) | 0.0037 |
|  | PFTrDA | 0.93 (0.89–0.97) | 0.0010 | 0.94 (0.90–0.99) | 0.0115 |
|  | PFHxS | 1.03 (0.98–1.09) | 0.2808 | 1.02 (0.96–1.09) | 0.4951 |
|  | PFOS | 0.92 (0.85–0.99) | 0.0271 | 0.91 (0.83–1.00) | 0.0556 |

Note: PFAS, per- and polyfluoroalkyl substances; PFDA, perfluorodecanoic acid; PFHxS, perfluorohexane sulphonic acid; PFNA, perfluorononanoic acid; PFOA, perfluorooctanoic acid; PFOS, perfluorooctane sulphonic acid; PFTrDA, perfluorotridecanoic acid; PFUnA, perfluoroundecanoic acid. Estimates show odds ratios for functional constipation per doubling of PFAS concentrations. Models included maternal age, maternal body mass index, parity, maternal smoking during pregnancy, maternal education, household income, and Study Area as confounders. A Bonferroni correction was applied to adjust for multiple comparisons, yielding a significance threshold of P < 0.0035. PFAS concentrations were transformed using the base-2 logarithm.

# Table S15. Associations between maternal PFAS concentrations and functional constipation in children at ages 3 and 4 years in the Japan Environment and Children’s Study, using modified Poisson models (n=17,686)

|  |  | **Crude** |  | **Adjusted** |  |
| --- | --- | --- | --- | --- | --- |
| **Outcome assessment** | **PFAS** | **Risk ratio (95% CI)** | **p** | **Risk ratio (95% CI)** | **p** |
| At 3 years | PFOA | 1.04 (1.00–1.09) | 0.0488 | 1.05 (0.99–1.11) | 0.0795 |
|  | PFNA | 0.98 (0.93–1.04) | 0.6126 | 1.00 (0.94–1.07) | 0.8992 |
|  | PFDA | 0.96 (0.91–1.02) | 0.1610 | 0.99 (0.93–1.06) | 0.8732 |
|  | PFUnA | 0.93 (0.87–0.99) | 0.0187 | 0.97 (0.90–1.03) | 0.3171 |
|  | PFTrDA | 0.99 (0.95–1.02) | 0.3911 | 1.01 (0.97–1.04) | 0.7482 |
|  | PFHxS | 1.04 (1.00–1.09) | 0.0298 | 1.05 (1.00–1.10) | 0.0497 |
|  | PFOS | 0.98 (0.92–1.04) | 0.4409 | 1.01 (0.94–1.08) | 0.8179 |
| At 4 years | PFOA | 1.09 (1.05–1.15) | 0.0001 | 1.11 (1.05–1.18) | 0.0005 |
|  | PFNA | 1.03 (0.97–1.09) | 0.3841 | 1.03 (0.96–1.11) | 0.4185 |
|  | PFDA | 0.94 (0.89–1.00) | 0.0683 | 0.95 (0.89–1.03) | 0.2095 |
|  | PFUnA | 0.89 (0.83–0.95) | 0.0007 | 0.90 (0.83–0.97) | 0.0061 |
|  | PFTrDA | 0.94 (0.90–0.97) | 0.0002 | 0.95 (0.91–0.98) | 0.0049 |
|  | PFHxS | 1.05 (1.01–1.10) | 0.0224 | 1.05 (0.99–1.10) | 0.0845 |
|  | PFOS | 0.94 (0.89–1.01) | 0.0759 | 0.94 (0.87–1.02) | 0.1451 |

Note: PFAS, per- and polyfluoroalkyl substances; PFDA, perfluorodecanoic acid; PFHxS, perfluorohexane sulphonic acid; PFNA, perfluorononanoic acid; PFOA, perfluorooctanoic acid; PFOS, perfluorooctane sulphonic acid; PFTrDA, perfluorotridecanoic acid; PFUnA, perfluoroundecanoic acid. Estimates show risk ratios for functional constipation per doubling of PFAS concentrations. Adjusted models included maternal age, maternal body mass index, parity, maternal smoking during pregnancy, maternal education, household income, maternal dietary habits, and Study Area as confounders. A Bonferroni correction was applied to adjust for multiple comparisons, yielding a significance threshold of P < 0.0035. PFAS concentrations were transformed using the base-2 logarithm.

**
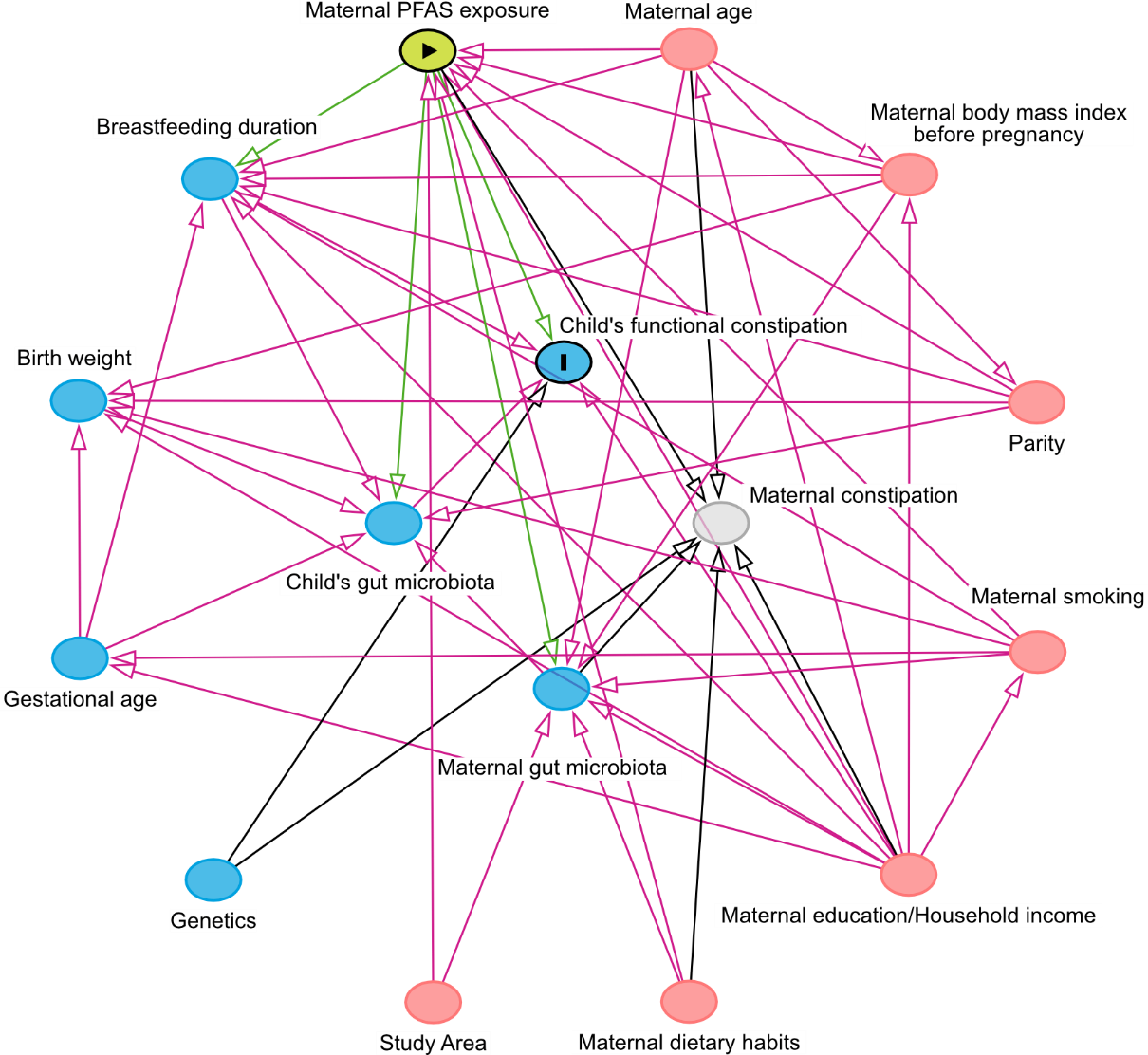
**

# Figure S1. Directed acyclic graphs illustrating the hypothesized association between maternal PFAS exposure and child functional constipation. The corresponding code is provided in Appendix S1. Refer to the accompanying code for specific relationships. PFAS, per- and polyfluoroalkyl substances;
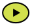
, exposure;
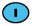
, outcome;
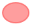
, ancestor of exposure and outcome;
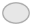
, unobserved variable;
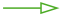
, causal path;
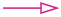
, bias path.

# Appendix S1. DAGitty model code for the directed acyclic graphs illustrating the hypothesized association between maternal PFAS exposure and child functional constipation. The corresponding graph is shown in Figure S1.

dag {

bb="-5,-5,5,5"

"Child's_functional_constipation" [outcome,pos="0.021,-0.997"]

"Child's_gut_microbiota" [pos="-1.009,-0.023"]

"Maternal_education/Household_income" [pos="2.112,2.109"]

Birth_weight [pos="-2.920,-0.764"]

Breastfeeding_duration [pos="-2.123,-2.108"]

Genetics [pos="-2.102,2.141"]

Gestational_age [pos="-2.911,0.798"]

Maternal_PFAS_exposure [exposure,pos="-0.801,-2.886"]

Maternal_age [pos="0.782,-2.896"]

Maternal_body_mass_index_before_pregnancy [pos="2.118,-2.135"]

Maternal_constipation [pos="0.975,-0.023"]

Maternal_dietary_habits [pos="0.782,2.880"]

Maternal_gut_microbiota [pos="0.009,0.982"]

Maternal_smoking [pos="2.895,0.759"]

Parity [pos="2.889,-0.753"]

Study_Area [pos="-0.770,2.883"]

"Child's_gut_microbiota" -> "Child's_functional_constipation"

"Maternal_education/Household_income" -> "Child's_functional_constipation"

"Maternal_education/Household_income" -> Birth_weight

"Maternal_education/Household_income" -> Breastfeeding_duration

"Maternal_education/Household_income" -> Gestational_age

"Maternal_education/Household_income" -> Maternal_PFAS_exposure

"Maternal_education/Household_income" -> Maternal_age

"Maternal_education/Household_income" -> Maternal_body_mass_index_before_pregnancy

"Maternal_education/Household_income" -> Maternal_constipation

"Maternal_education/Household_income" -> Maternal_gut_microbiota

"Maternal_education/Household_income" -> Maternal_smoking

Birth_weight -> "Child's_gut_microbiota"

Breastfeeding_duration -> "Child's_functional_constipation"

Breastfeeding_duration -> "Child's_gut_microbiota"

Genetics -> "Child's_functional_constipation"

Genetics -> Maternal_constipation

Gestational_age -> "Child's_gut_microbiota"

Gestational_age -> Birth_weight

Gestational_age -> Breastfeeding_duration

Maternal_PFAS_exposure -> "Child's_functional_constipation"

Maternal_PFAS_exposure -> "Child's_gut_microbiota"

Maternal_PFAS_exposure -> Breastfeeding_duration

Maternal_PFAS_exposure -> Maternal_constipation

Maternal_PFAS_exposure -> Maternal_gut_microbiota

Maternal_age -> Breastfeeding_duration

Maternal_age -> Maternal_PFAS_exposure

Maternal_age -> Maternal_body_mass_index_before_pregnancy

Maternal_age -> Maternal_constipation

Maternal_age -> Maternal_gut_microbiota

Maternal_age -> Parity

Maternal_body_mass_index_before_pregnancy -> Birth_weight

Maternal_body_mass_index_before_pregnancy -> Breastfeeding_duration

Maternal_body_mass_index_before_pregnancy -> Maternal_PFAS_exposure

Maternal_body_mass_index_before_pregnancy -> Maternal_gut_microbiota

Maternal_dietary_habits -> Maternal_PFAS_exposure

Maternal_dietary_habits -> Maternal_constipation

Maternal_dietary_habits -> Maternal_gut_microbiota

Maternal_gut_microbiota -> "Child's_gut_microbiota"

Maternal_gut_microbiota -> Maternal_constipation

Maternal_smoking -> Birth_weight

Maternal_smoking -> Breastfeeding_duration

Maternal_smoking -> Gestational_age

Maternal_smoking -> Maternal_PFAS_exposure

Maternal_smoking -> Maternal_gut_microbiota

Parity -> "Child's_gut_microbiota"

Parity -> Birth_weight

Parity -> Breastfeeding_duration

Parity -> Maternal_PFAS_exposure

Study_Area -> Maternal_PFAS_exposure

Study_Area -> Maternal_gut_microbiota

}
